# Supplementary material for: The effect of wheat genotype on the microbiome is more evident in roots and varies through time
Source: ISME Commun. 2023 Apr 19;3:32. doi: 10.1038/s43705-023-00238-4 (PMC10115884; doi:10.1038/s43705-023-00238-4)
Supplement: Supplementary file 2 — Table S2 [file 43705_2023_238_MOESM2_ESM.docx]

**Supplementary Table S1.** Anova tests and Tukey HSD post-hoc tests for the effect of genotype on the relative abundance of bacterial phylum/classes in the roots based on the 16S rRNA gene amplicon dataset.

|  |  | **2013** | **2013** | **2014** | **2015** | **2015** |
| --- | --- | --- | --- | --- | --- | --- |
|  |  | **SE** | **DD** | **SE** | **DD** | **DD** |
|  |  | **Gamma** | **Actino** | **Gamma** | **Alpha** | **Gamma** |
| *Anova* |  |  |  |  |  |  |
| F |  | 2.65 | 2.86 | 5.63 | 9.94 | 3.44 |
| P |  | 0.0373 | 0.0257 | 0.006 | 1.68 x 10^-5^ | 0.0113 |
|  |  |  |  |  |  |  |
| *Tukey HSD* | *Genotype* |  |  |  |  |  |
| *Triticum turgidum* | Pelissier (1929) | a | b | ab | abc | ab |
|  | Strongfield (2004) | ab | ab | a | ab | ab |
|  | CDC Verona (2008) | ab | a | ab | a | b |
|  | CDC Stanley (2009) | ab | ab | b | d | ab |
| *Triticum aestivum* | Red Fife (1845) | ab | ab | ab | abc | ab |
|  | Marquis (1911) | ab | ab | b | cd | ab |
|  | CDC Teal (1991) | ab | ab | b | d | a |
|  | AC Barrie (1994) | b | ab | ab | cd | ab |
|  | Lillian (2003) | ab | ab | b | d | ab |
|  | CDC Kernen (2009) | ab | ab | ab | bcd | a |
