# Supplementary material for: The effect of wheat genotype on the microbiome is more evident in roots and varies through time
Source: ISME Commun. 2023 Apr 19;3:32. doi: 10.1038/s43705-023-00238-4 (PMC10115884; doi:10.1038/s43705-023-00238-4)
Supplement: Supplementary file 3 — Table S3 [file 43705_2023_238_MOESM3_ESM.docx]

**Supplementary Table S2.** Anova tests and Tukey HSD post-hoc tests for the effect of genotype on the relative abundance of fungal phyla in the roots based on the ITS region amplicon dataset.

|  |  | **2013** | **2013** |
| --- | --- | --- | --- |
|  |  | **SE** | **SE** |
|  |  | **Ascomycota** | **Basidiomycota** |
| *Anova* |  |  |  |
| F |  | 3.13 | 2.58 |
| P |  | 0.01 | 0.03 |
|  |  |  |  |
| *Tukey HSD* | *Genotype* |  |  |
| *Triticum turgidum* | Pelissier (1929) | ab | a |
|  | Strongfield (2004) | ab | a |
|  | CDC Verona (2008) | a | a |
|  | CDC Stanley (2009) | ab | a |
| *Triticum aestivum* | Red Fife (1845) | ab | a |
|  | Marquis (1911) | b | a |
|  | CDC Teal (1991) | ab | a |
|  | AC Barrie (1994) | ab | a |
|  | Lillian (2003) | ab | a |
|  | CDC Kernen (2009) | ab | a |
