# Supplementary material for: The effect of wheat genotype on the microbiome is more evident in roots and varies through time
Source: ISME Commun. 2023 Apr 19;3:32. doi: 10.1038/s43705-023-00238-4 (PMC10115884; doi:10.1038/s43705-023-00238-4)
Supplement: Supplementary file 4 — Table S4 [file 43705_2023_238_MOESM4_ESM.docx]

**Supplementary Table S3.** Anova tests and Tukey HSD post-hoc tests for the effect of genotype on the relative abundance of bacterial phylum/classes in the roots based on the cpn60 gene amplicon dataset.

|  |  | **2013** | **2013** | **2014** | **2016** | **2016** | **2016** | **2016** |
| --- | --- | --- | --- | --- | --- | --- | --- | --- |
|  |  | **DD** | **DD** | **DD** | **DD** | **DD** | **DD** | **DD** |
|  |  | **Acido** | **Actino** | **Acido** | **Acido** | **Alpha** | **Bacteroid** | **Verruc** |
| *Anova* |  |  |  |  |  |  |  |  |
| F |  | 2.51 | 3.15 | 2.47 | 4.59 | 3.44 | 4.75 | 2.79 |
| P |  | 0.0487 | 0.0199 | 0.0489 | 0.006 | 0.0217 | 0.005 | 0.045 |
|  |  |  |  |  |  |  |  |  |
| *Tukey HSD* | *Genotype* |  |  |  |  |  |  |  |
| *Triticum turgidum* | Pelissier (1929) | a | b | ab | ab | ab | b | a |
|  | Strongfield (2004) | a | ab | ab | ab | ab | b | a |
|  | CDC Verona (2008) | a | a | ab | ab | ab | ab | a |
|  | CDC Stanley (2009) | a | ab | a | ab | ab | b | a |
| *Triticum aestivum* | Red Fife (1845) | a | ab | ab | ab | a | b | a |
|  | Marquis (1911) | a | ab | ab | ab | ab | a | a |
|  | CDC Teal (1991) | a | ab | b | a | ab | b | a |
|  | AC Barrie (1994) | a | ab | ab | ab | ab | b | a |
|  | Lillian (2003) | a | ab | ab | a | b | b | a |
|  | CDC Kernen (2009) | a | ab | ab | ab | b | b | a |
